# Evaluation of controls, quality control assays, and protocol optimisations for PacBio HiFi sequencing on diverse and challenging samples

**DOI:** 10.3389/fgene.2024.1505839

**Published:** 2025-01-06

**Authors:** Iraad F. Bronner, Emma Dawson, Naomi Park, Olaf Piepenburg, Michael A. Quail

**Affiliations:** Wellcome Sanger Institute (WT), Hinxton, United Kingdom

**Keywords:** PacBio, HiFi, quality controls, sequencing, long read, Earth BioGenome project

## Abstract

The Darwin Tree of Life (DToL) project aims to generate high-quality reference genomes for all eukaryotic organisms in Britain and Ireland. At the time of writing, PacBio HiFi reads are generated for all samples using the Sequel IIe systems by the Wellcome Sanger Institute’s Scientific Operations teams, however we expect lessons from this work to apply directly to the Revio system too, as core principles of SMRT sequencing remain the same. We observed that HiFi yield is highly variable for DToL samples. We have investigated what drives this variation, and potential mitigations. To support these investigations a number of controls were evaluated to ensure that the library and sequencing preparation procedures, reagents, consumables, and Sequel IIe instruments, were performing as expected. Our findings support that a primary factor driving variability in HiFi yield is the quality of the DNA prior to library construction, e.g., purity, size, and damage. We investigated whether quality assessment assays could link measurable DNA damage or purity to sequencing yield. Some correlation could be established, however no assay was predictive of sequencing yield for all samples, indicating that the variability is driven by multiple factors that may interact. We demonstrate that contaminants present in some samples are the cause of very low HiFi yield, and show that these contaminants can negatively affect the PacBio internal sequencing control and samples multiplexed on the same SMRT Cell. We found that consistently high yields could be obtained if an amplification workflow was utilised, namely PacBio’s ultra-low input library preparation protocol.

## Introduction

The Darwin Tree of Life (DToL) project is an ambitious biodiversity genomics project which aims to produce high-quality reference genomes for all known eukaryotic species in Britain and Ireland. The project is a collaboration between biodiversity organisations and genomics institutes in which the Wellcome Sanger Institute (Sanger) has a leading role (Darwin Tree of Life Project Consortium, 2022). DToL is one of many projects across the globe contributing to the Earth BioGenome Project, an endeavour to sequence the genomes of all of Earth’s eukaryotic biodiversity (Lewin et al., 2018). The initiative aims to increase our understanding of Earth’s biodiversity including but not limited to biological processes such as adaptation, the reasons for species extinctions, and the importance of individual species to functioning ecosystems (Lewin et al., 2018). All the data produced for DToL is published and freely available to researchers (Darwin Tree of Life, 2022). All samples processed at Sanger are sequenced in-house by Sanger’s Sequencing Operations teams. In collaboration with the Tree of Life (ToL) programme, Sequencing Operations teams have evaluated controls, quality assessment assays, and protocol optimisations for these diverse and challenging samples (Darwin Tree of Life Project Consortium, 2022).

To produce high quality genomes, capturing all types of genetic variation and repeat structures; high-quality, high-throughput long-read sequencing is required. At the time of writing, the approach utilised for the DToL project is predominantly Single Molecule, Real-Time (SMRT) sequencing (Eid et al., 2009), using the circular consensus sequencing (CCS) mode, from Pacific Biosciences (PacBio, CA, United States). SMRTbell libraries are prepared by ligating adaptors to double-stranded DNA creating a circular template. Primer and polymerase molecules are bound to the library before it is loaded onto the Sequel IIe system. Sequencing occurs on a SMRT Cell, each containing millions of wells called zero-mode waveguides (ZMWs). The SMRTbell template is immobilised at the bottom of the ZMW. SMRT sequencing is based on the observation of the temporal order of fluorescently labelled nucleotide incorporations during DNA synthesis by a polymerase molecule (Eid et al., 2009). CCS is used to generate highly accurate long reads called HiFi reads. The work described here predates PacBio’s Revio system and was completed using the Sequel IIe system. However, we expect that lessons learned from this work will translate directly to the Revio system, since the basic principles of SMRT sequencing and SMRTbell library preparation remain unchanged.

Since the adoption of the Sequel II system, Sanger’s HiFi yields for DToL samples have ranged from 0–38 Gb per 8M SMRT Cell (PacBio states that 30 Gb HiFi Yield can be generated from one 8M SMRT Cell). The majority of the species sequenced throughout the first year of the DToL project had a genome size of less than 1 Gigabase (Gb), meaning only one 8M SMRT Cell yielding more than 25 Gb of HiFi data was required to produce sufficient coverage (25x) for high-quality genome assembly. This resulted in few sequencing libraries that required additional SMRT Cells to be run in order to complete the genome. However, due to minimum coverage requirements for high quality genome assembly, samples generating less than 15 Gb (less than 15x coverage for a 1 Gb genome) of HiFi data from one 8M SMRT Cell will require at least a second SMRT Cell to be run to achieve 25x coverage. This has a cost implication, while also reducing the total potential sequencing capacity of our sequencing fleet. The diversity of these samples, each with different biology and metabolites, makes establishing informative quality control assays very challenging. At the time of writing, there is no single quality assessment assay available to reliably predict PacBio sequencing results.

Therefore, the purpose of this work was to increase our understanding of what factors drive variability in HiFi yield. In this work we evaluated a number of quality assessment assays and their ability to predict sequencing yield, and investigated methods to increase HiFi yields for challenging samples.

## Results

### Evaluation of process controls

**FIGURE 1:**
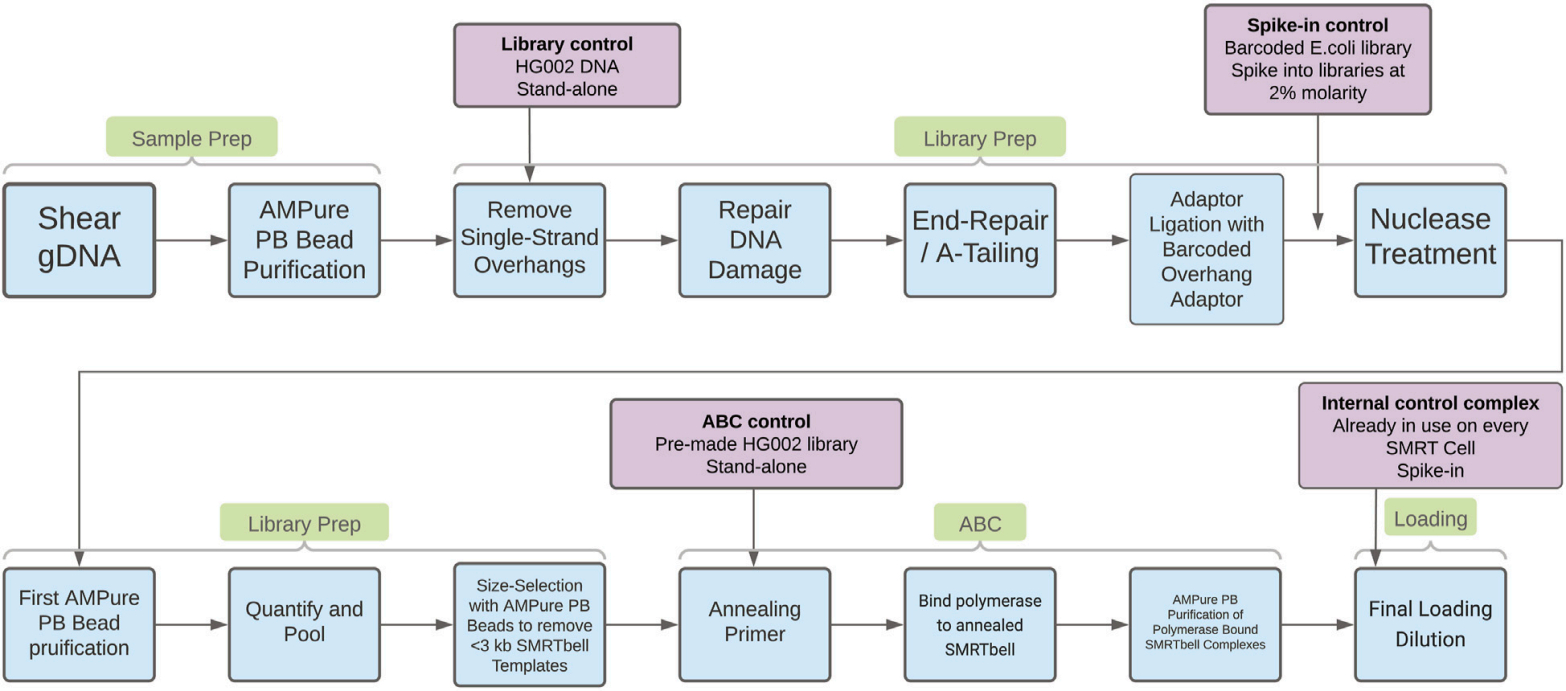
In blue boxes, process steps in the “Preparing HiFi Libraries from Low DNA Input Using SMRTbell Express Template Prep Kit 2.0” protocol. In purple boxes, details of the process controls evaluated in this work. The arrows from the purple boxes indicate where the control is introduced in the workflow.

To support investigations into the drivers of sequencing yield variability, three different process controls were evaluated. These controls are designed to help understand quality issues in different stages of PacBio’s “Preparing HiFi Libraries from Low DNA Input Using SMRTbell Express Template Prep Kit 2.0” protocol (from PacBio, CA, United States; see Figure 1 for an overview of the workflow and controls). With these controls it is possible to establish how much variability is introduced by the methods, reagents, and instruments in use at Sanger, and to provide insight into the drivers of low HiFi yield. The three controls we will discuss below are the “library control,” the “spike-in control,” and the “ABC control.”

#### Library control

The “Library control” is made from DNA extracted from a human cell line (HG002). DNA was fragmented in bulk using the Megaruptor 3 (Diagenode SA, Belgium), purified using a 0.6X AMPure PB bead (PacBio, CA, United States) clean-up, divided into aliquots, and frozen at −20°C. One aliquot was included in each batch of samples undergoing library creation to confirm that the reagents and method used are performing well.

The loss of DNA during the first purification step of the SMRTbell templates after nuclease treatment (see Figure 1) showed higher variability for DToL samples compared to the library control (see Supplementary Figure 1). The DNA recovery for library control samples was always >30%, whereas for DToL samples this was as low as 15%. The nuclease treatment removes damaged or un-ligated SMRTbell templates, therefore higher loss during this step could indicate the presence of DNA damage or contaminants which inhibit adaptor ligation resulting in un-ligated templates.

Recovery of the library control DNA from the size selective diluted AMPure clean-up at the end of library preparation (see Figure 1) ranged from 45% to 80% (see Supplementary Figure 1). This variation could be due to the nature of the size selective clean up, the volumes need to be very accurate in order to achieve the exact cut off intended. Even slightly imprecise volumes will affect the size selection and consequently the recovery. This could be addressed by automating this purification step. Some variation could also be a result of the procedure to dilute the AMPure PB beads. This could be addressed by introducing batch control for the beads used in this step, and thorough testing of batches.

All library controls passed library preparation, see methods section for further details. Sequencing yields did not correlate with library preparation batches.

#### Spike-in control - Distinguishing between DNA damage and impurities

The “spike-in control” is made from DNA extracted from *E. coli* K12, which was taken through the protocol “Preparing HiFi Libraries from Low DNA Input Using SMRTbell Express Template Prep Kit 2.0” up to and including adaptor ligation (see Figure 1). This DNA was then purified using a 1x AMPure PB clean-up, divided into aliquots, and frozen at −20°C for future use.

The Tree of Life (ToL) teams at Sanger created a panel of samples representing a diverse range of species and sample types which can be used for research and development work (see Supplementary Figure 2 for further details on these species and others mentioned below). The *E. coli* control was spiked into seven ToL R&D panel species libraries, prior to nuclease treatment, at 2% molarity. In principle, if the spike-in control sequences well but the sample does not, this indicates that the sample contains unrepaired DNA damage or impurities inhibiting the adaptor ligation. This is because the spike-in control acts as a control for all process steps after adaptor ligation. If the spike-in DNA and the sample DNA both do not sequence well, but the PacBio internal control complex (ICC) does sequence as expected, this indicates the presence of impurities which are inhibiting the ABC reaction. The ICC is a PacBio supplied control library used to differentiate between instrument/consumable related performance issues, and sample-related issues. This control library is a pre-assembled complex of adapter-ligated fragment, sequencing primer, and polymerase. If the spike-in control DNA, the sample DNA, and the ICC all fail to sequence, this indicates either a consumables/system failure or the presence of a very strong contaminant inhibiting sequencing.

**FIGURE 2:**
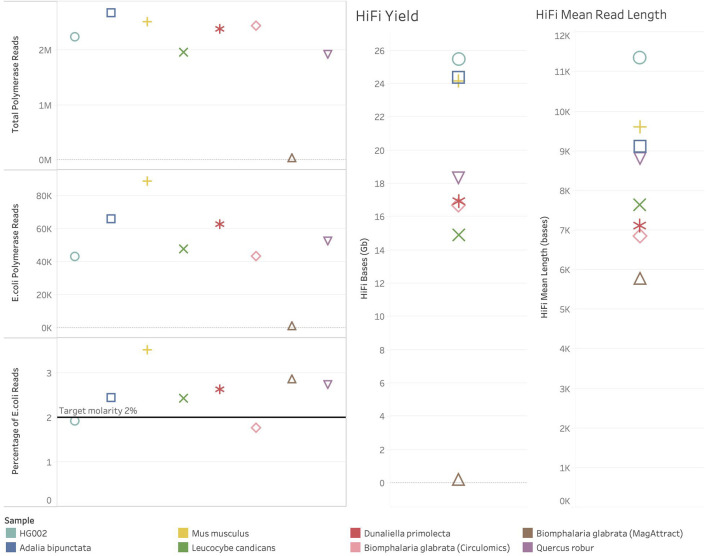
Sequencing metrics for ToL R&D samples with spike-in control present. Colour shows detail about sample. Shapes show detail about sample, HG002 – circle, *Adalia bipunctata*–square, *Mus musculus*–plus sign, *Leucocybe candicans*–cross, *Dunaliella primolecta*–star, *Biomphalaria glabrata* (Circulomics) – diamond, *Biomphalaria glabrata* (MagAttract) – triangle, *Quercus robu*r–inverted triangle. On the left are plots showing the number of polymerase reads generated in each sequencing run. At the top left, Total Polymerase Reads generated from each sequencing run. In the middle left, the number of Polymerase reads generated from the *Escherichia coli* spike-in control sequenced. At the bottom left, the percentage of total polymerase reads that are *Escherichia coli* reads. On the right-hand side of the figure are HiFi metrics for yield and mean read length.

In one of our species, *Biomphalaria glabrata* (a species of freshwater snail) DNA extracted using a Qiagen MagAttract kit (QIAGEN Ltd., United Kingdom), failed to sequence efficiently, generating only 6.9 Gb of total bases (total bases is calculated by multiplying the number of Productive (P1) ZMWs by the mean polymerase read length). Similarly, the number of successful reads and HiFI yield were also poor (see Figure 2). In this case, the spike-in control and sample both failed to sequence. The internal control complex (ICC) also failed to sequence. This was observed with all *Biomphalaria glabrata* MagAttract extracted DNA sequenced at the time of writing. This indicates that there is a contaminant present inhibiting PacBio’s DNA polymerase. In contrast, *Biomphalaria glabrata* DNA extracted using a Circulomics kit (PacBio, MD, United States) sequenced well, generating 492 Gb of total bases. For this sample polymerase read count and HiFi yield were normal (see Figure 2). This suggests that the problematic contaminant present in the DNA isolated using the MagAttract kit is absent when the DNA is isolated using the Circulomics NanoBind Animal Big Tissue Kit (PacBio, MD, United States).

All other ToL R&D panel samples sequenced generated over 380 Gb total bases and the number of reads and HiFI yield were also normal (See Figure 2). The percentage of spike-in control reads was close to 2% for all species. The samples, excluding *Biomphalaria glabrata* (MagAttract), show expected metrics for Total Bases, Mean Polymerase Read Length, productivity metrics (P1% ZMW), Local Base Rate, and Internal Control Complex Read Length (bp). However, four samples still generated <20 Gb of HiFi data (see Figure 2), and no sample generated >25 Gb on a single SMRT Cell. Given that the aforementioned metrics are all as expected, this is likely due to the insert size reflected by HiFi Mean Read Length in Figure 2. The HiFi mean read length is below 10 Kb for all samples (for optimal HiFi yield PacBio recommends an insert size of 15–20 Kb). Short fragments present in these libraries are limiting the HiFi yield which can be generated.

#### ABC control

The “ABC control” is a library generated using DNA extracted from the human HG002 cell line. The library was divided into aliquots and frozen at −20°C to mitigate for any effect of multiple freeze thaw cycles. Sequencing complex creation consists of three steps; primer annealing, polymerase binding and complex clean-up, known as the ABC reactions (see Figure 1). A complex was made from the ABC control alongside other samples to assess the performance of the reagents and method, and to establish how much variation is introduced by these protocol steps. This control could also be used to assess variability between different SMRT Cells, and between different sequencing instruments.

**FIGURE 3:**
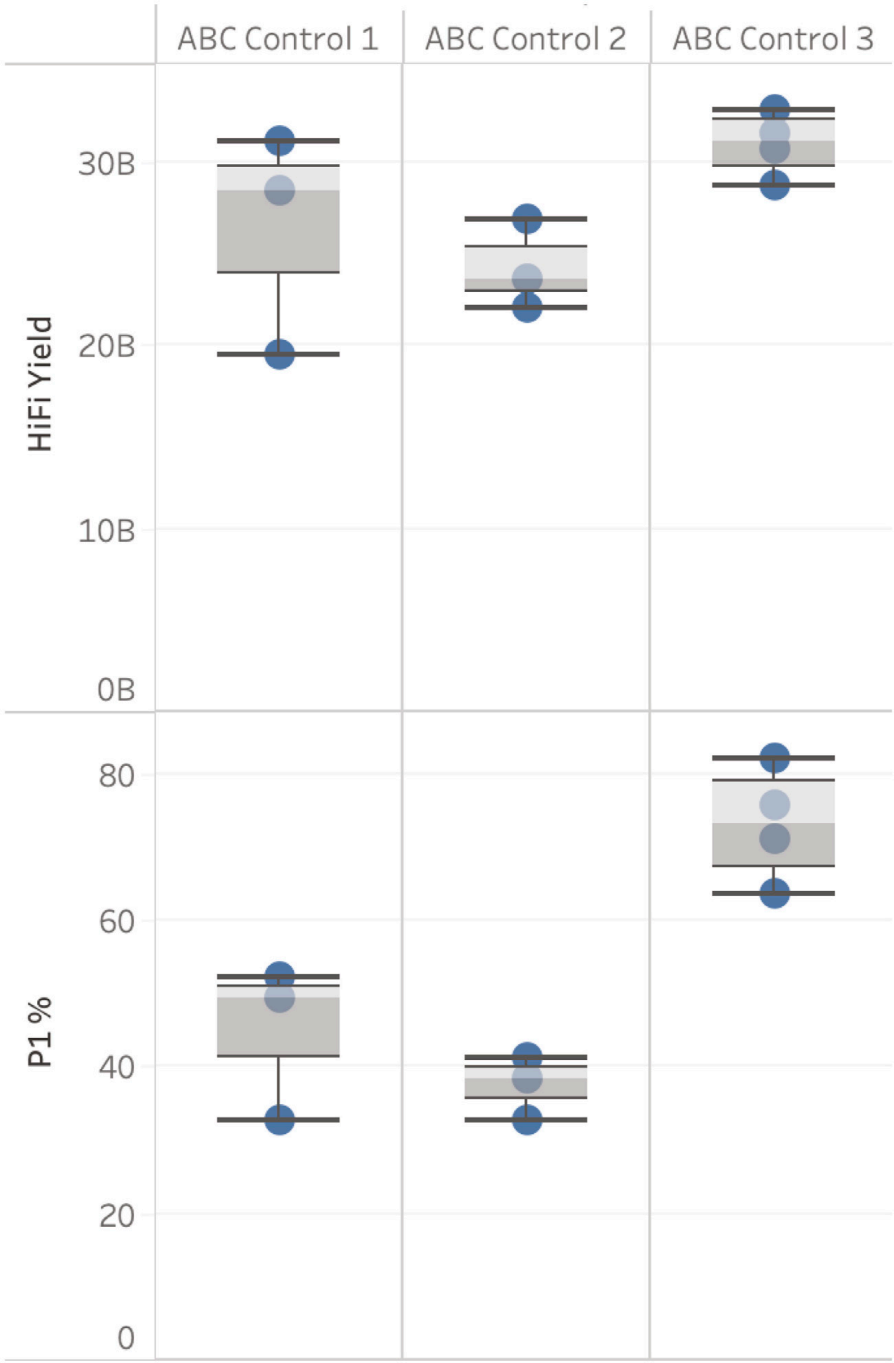
ABC control results. HiFi yield (Gb) and P1 (%), broken down by ABC Control. Data is filtered for each ABC Control to only show runs with the same sequencing run conditions including sample on plate loading concentrations (OPLC), pre-extension time, and binding kit.

Three ABC controls were used as part of this evaluation (see Figure 3). HiFi yield from three sequencing runs for ABC Control 1 showed a coefficient of variation (CV) of 23% (sequenced in October 2021). One of the three SMRT Cells generated a yield of 19.4 Gb, see Figure 3. This result indicates that either the complex creation or the SMRT Cell used caused this variability in yield. However, this result also demonstrates that when a high-quality sample has poor loading metrics (32.77% ZMWs were categorised as “P1” in this case, “P1” being the percentage of ZMWs that are productive, PacBio recommends 60%–70% P1 is optimal) yields close to 20 Gb can still be generated. This supports a hypothesis that the level of variability observed for DToL samples is not solely explained by variability in complex creation or SMRT Cell performance.

ABC Control 2 (sequenced on three SMRT cells) and 3 (sequenced on four SMRT Cells) (sequenced November 2021 - March 2022) had a CV <10%. The four sequencing results for ABC Control 3 were generated on four different Sequel IIe systems and were prepared and loaded by four different users.

### Inhibition of reactions during complex creation (ABC)

SMRTbell libraries were generated for four ToL R&D panel species using the ‘Preparing whole genome libraries using SMRTbell prep kit 3.0’ protocol (from PacBio, CA, United States). These libraries were normalised to the same molarity and then combined into four separate pools, each with different ratios of each sample, shown in Table 1.

**TABLE 1:** Pooling strategy for libraries. AdaBipu: *Adalia bipunctata* library, QueRobu: *Querqus Robur* library, MusMuc: *Mus musculus* library, and MetZobe: *Metschnikowiella zobelli l*ibrary.

Sequencing pool	Ratios of each SMRTbell library			
	50%	35%	10%	5%
Pool 1	AdaBipu	QueRobu	MusMuc	MetZobe
Pool 2	MetZobe	AdaBipu	QueRobu	MusMuc
Pool 3	MusMuc	MetZobe	AdaBipu	QueRobu
Pool 4	QueRobu	MusMuc	MetZobe	AdaBipu

Each pool then went through complex creation (ABC) and was sequenced on one 8M SMRT Cell. The sequencing results are shown in Table 2. The HiFi yield for the pools correlates negatively with the quantity of *Metschnikowiella zobelli* library in the pool. The internal control complex sequenced as expected. This data supports a hypothesis that a contaminant present in the *M. zobelli* library is inhibiting the annealing of the primer or binding of the polymerase to the template DNA of the entire pool during complex creation (ABC). When *M. zobelli* is present in higher quantities the observed inhibition is stronger, resulting in less productive templates for sequencing. This is different to the previous observations made for *Biomphalaria glabrata,* where contaminants inhibited the polymerase during sequencing as shown by the inhibition of the internal sequencing control which is a pre-made complex.

**TABLE 2:** Percentage of de-plexed reads associated with each sample in each pool, and total Hifi Yield from each pool. Colour indicates the same library shown in Table 1. AdaBipu shown in pink, QueRobu shown in blue, MusMuc shown in green, and MetZobe shown in yellow.

Sequencing pool	Split				HiFi yield (Gb)
	50%	35%	10%	5%	
Pool 1	47.4	30.7	11	7	29
Pool 2	47.7	32.7	10.4	6.3	4.13
Pool 3	45.7	34.12	10.8	6.2	3.35
Pool 4	44.5	31.6	13.7	7.2	16.8

### Evaluation of DNA quality assessment assays for PacBio sequencing

One challenging aspect of biodiversity sequencing projects in which multiple species and sample types are being sequenced, is a lack of assays which can predict how well a sample will sequence. For some sequencing technologies, low-cost, low throughput, flow cells are available which can be used for development experiments. For example, the Flongle flow cell, produced by Oxford Nanopore Technologies, or Illumina’s MiSeq. There is currently no equivalent for PacBio sequencing. We developed and tested a number of non-sequencing analytical assays with the aim of identifying possible drivers of variable HiFi yields. These assays fell into three classes:

1. Analysis of DNA integrity (i.e., average fragment size post DNA isolation)
2. Analysis of DNA damage or “amplifyability” (e.g., nicks, nucleotide base damage and crosslinks)
3. Analysis of DNA purity

When evaluating these assays, we utilised the ToL R&D panel, for which we have plenty of available material. Samples from these species are used routinely for research and development work. The panel contains a range of samples, some of which typically generate high sequencing yield and some which are more challenging (see Table 3 for species details).

**TABLE 3:** HiFi yield in Gigabases from one 8M SMRT Cell from representative genomic DNA samples prepared from the ToL R&D panel. Total ZMW occupancy is total active ZMW per 8M chip to show total loading percentage (P1 plus P2).

Species	HiFi yield (Gb)	Total ZMW occupancy (P1 + P2 %)
*Huperzia selago* (Huperzia selago, 2020)	16	82%
*Geum rivale* (Geum rivale, 2024)	8	46%
*Tholera decimalis* (Boyes et al., 2023)	9	40%
*Lathraea squamaria* (Lathraea squamaria, 2024)	17	84%
*Adalia bipunctata* (Wellcome Sanger Institute Tree of Life programme and Wellcome Sanger Institute Scientific OperationsWellcome Sanger Institute Scientific Operations: DNA Pipelines collectiveTree of Life Core Informatics collectiveDarwin Tree of Life Consortium, 2022)	21	85%
*Biomphalaria glabrata* (Tree of Life QC, 2024a)	0	3%
*Dunaliella primolecta* (Rad-Menéndez et al., 2023)	14	79%
*Physella acuta* (Physella acuta, 2024)	7	37%
*Quercus robur* (Quercus robur, 2024)	12	79%
*Teleogryllus oceanicus* (Teleogryllus oceanicus, 2024)	20	78%

#### DNA integrity

The fragment size distribution of DNA samples is routinely measured at multiple points during the sample preparation workflow, this is performed using automated pulsed-field capillary electrophoresis, primarily the Femto Pulse system (from Agilent, CA, United States). This allows us to measure DNA fragment length after DNA extraction, shearing, and size selection during library preparation. The complex information contained in the Femto Pulse electropherogram can be simplified by obtaining a Genomic Quality Number (GQN) value. The GQN represents the proportion of fragments above a chosen threshold. For optimal sequencing results, PacBio recommends GQN values of >9 when the threshold is set at 10 Kb (PacBio, 2022) meaning that 90% of the DNA is larger than 10 Kb. We found that GQN alone was not a good predictor of either HiFi sequencing yield or productivity. We analysed a large data set of 293 DNA extracts and 248 sheared DNA samples, comparing the recommended GQN values after both DNA extraction and shearing with sequencing yield. This analysis found GQN alone was not a reliable QC metric to predict sequencing yield (see Supplementary Figure 3). *Geum rivale* for instance had a relatively high GQN of 8.5 but both sequencing yields and occupancy were low, while *Lathraea squamaria* and *Adalia bipunctata* had modest GQN values of 6.2 and 6.8 respectively but relatively high HiFi sequencing yield and occupancy. *Dunaliella primolecta* had a low GQN of only 4.8 yet sequenced well, especially with our low input library prep, and even though the freshwater snail *Biomphalaria glabrata* had a modest GQN of 6.3 it yielded virtually no sequencing data (see Table 3). Shorter fragment libraries will have reduced HiFi yield compared to larger fragment libraries when ZMW occupancy (P1) is the same, however a high GQN does not necessarily translate to high sequencing yield because of other issues at play. For example, our work evaluating the spike-in control clearly showed that some samples, e.g., *Biomphalaria glabrata,* contain an inhibitor impacting the DNA polymerase during sequencing. Samples which sequence poorly due to polymerase inhibitors may meet the recommended GQN but this will not translate to high sequencing yield. Therefore, while GQN can be a good predictor of size based HiFi conversion on clean (e.g., human cell line) DNA, it is not a good predictor of contamination-based variability.

#### DNA damage

Electrophoretic assays, such as the Femto Pulse system, measure fragment length and distribution of fragment lengths but do not report DNA damage, e.g., nicks, crosslinks or modifications. Nicked DNA will be indistinguishable from intact double stranded DNA of the same size. We therefore investigated assays to identify DNA damage.

##### Single stranded DNA nick assessment

**FIGURE 4:**
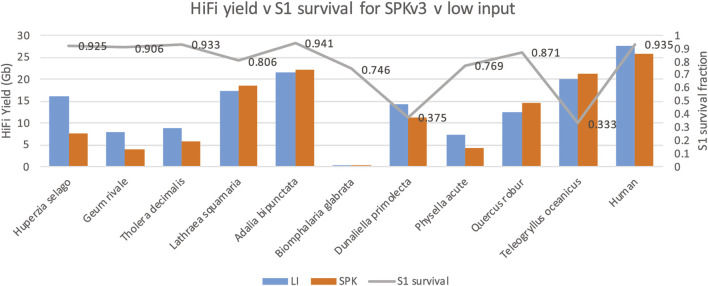
S1 survival ratio (grey line) compared to HiFi yield (Gb) for each genome obtained with both low input library method (blue, LI) and SPKv3 library prep (orange).

PacBio SMRT sequencing interrogates native single DNA molecules. Nicks in either DNA strand will result in the termination of DNA synthesis by the polymerase molecule and consequently the sequencing read, and therefore will result in reduced yield. The enzyme S1 nuclease is known to cut DNA strands opposite such nicks to create double strand breaks (Chaudhry and Weinfeld, 1995). To determine nick damage in our genomic DNA, we ran our samples on Femto Pulse before and after S1 nuclease digestion to assess DNA fragment sizes pre and post digestion and calculated an S1 survival ratio (defined as GQN digested/original undigested GQN). Intact DNA will have a survival ration of 1. For some samples, e.g., *D. primolecta* a large decrease in average DNA fragment size was seen after S1 nuclease digestion, indicating substantial presence of single strand DNA nicks in the native DNA. Again, however, these ratios could not predict low sequence yield (see Figure 4.) Some of the samples with high S1 survival ratio showed low yields, whereas some of the samples with low S1 survival ratio (*D. primolecta and Teleogryllus oceanicus)* showed average or high HiFi yields. This likely demonstrates that the damage repair step that is part of the PacBio library prep is adequately effective at repairing nicks present in damaged DNA.

##### qPCR to assess genomic DNA amplifiability

**FIGURE 5:**
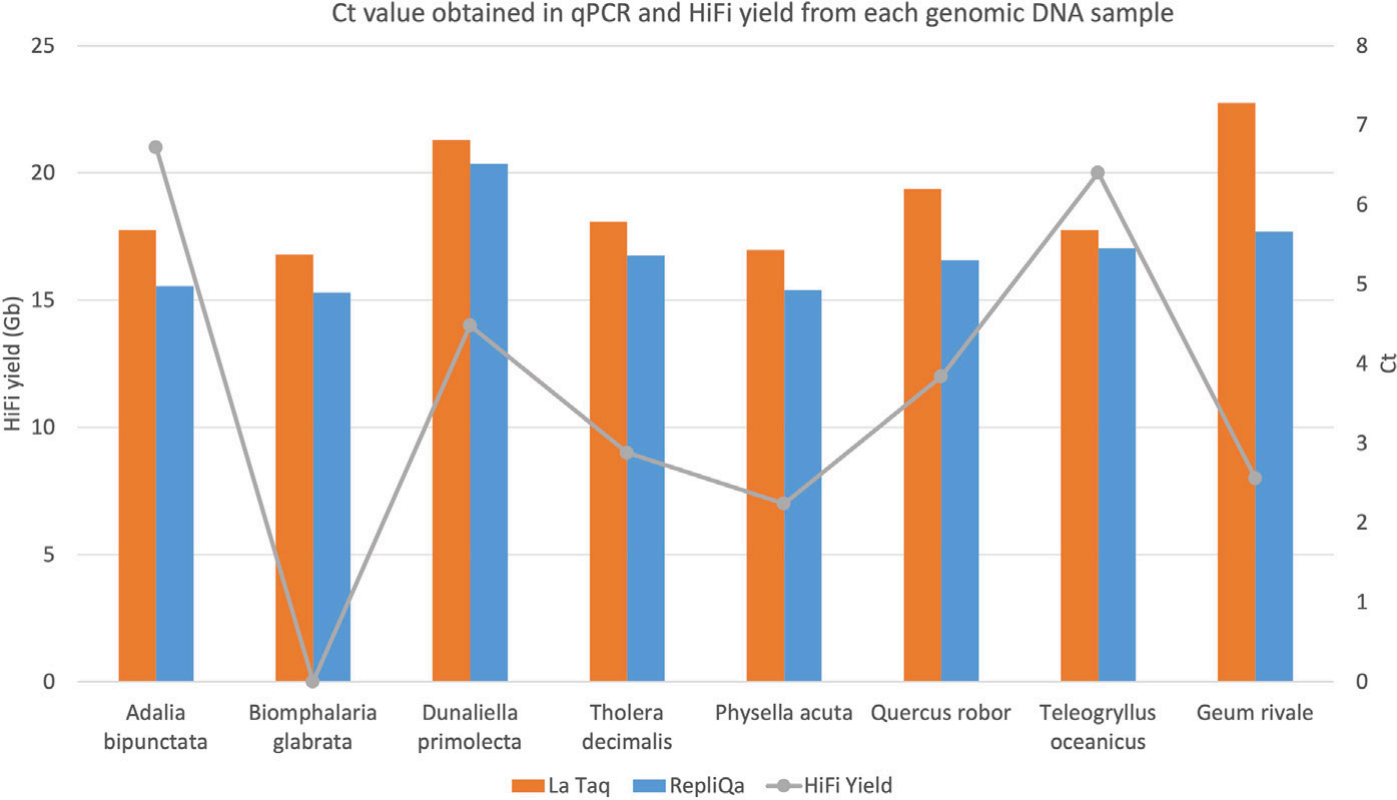
HiFi yield (Gb) and Ct value for each genome obtained with both TaKaRa LA Taq (orange) and Quantabio repliQa HiFi ToughMix (grey) PCR enzymes.

DNA damage interferes with PCR amplification (Sikorsky et al., 2007). To assay DNA damage on our samples by qPCR we attached Illumina adapters to 10 kb DNA fragments from each genomic DNA preparation and performed long fragment qPCR with 0.5 ng adapter ligated template. To avoid potential specific enzyme bias, we used both LA Taq DNA polymerase (Takara Bio Europe, France) and RepliQa HiFi ToughMix (Quantabio, MA, United States). As can be observed in Figure 5, Ct values were similar irrespective of HiFi yield and did not correlate to sequencing yield. Species which typically generate low HiFi yield such as *Biophalaria glabrata*, *G. rivale* and *Physella acuta*, gave similar Ct values when compared with species which typically generate good yields, such as *A. bipunctata* (Figure 5). These PCR enzymes however have been substantially optimised, to give good results, even in challenging conditions, meaning we hypothesised that maybe an enzyme more akin to PacBio’s sequencing enzyme might give more similar results.

##### Whole genome amplification (WGA)

**FIGURE 6:**
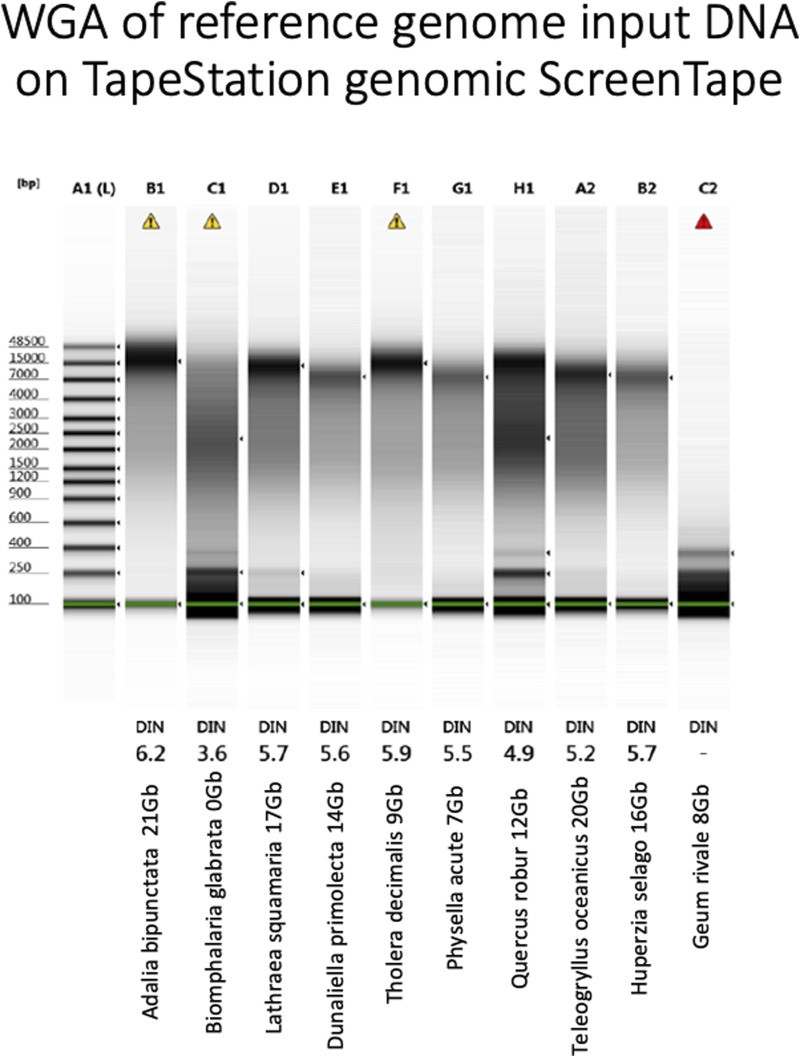
Agilent TapeStation genomic tape electropherogram (Agilent Technologies LDA United Kingdom Ltd., United Kingdom) of WGA products from each genomic DNA sample. Yield and name under each sample. DIN: DNA Integrity Number, the higher the better. Marker in DNA bases.

Since both WGA and PacBio’s SMRT sequencing use a phi-29 polymerase (Eid et al., 2009), we reasoned that efficiency of WGA as a QC metric on extracted DNA, may be a good proxy for sequencing performance and HiFi yield. To test this, we performed WGA on a 1 ng aliquot of each of our DNA samples and measured both yield (Table 4.) and size of amplified fragments (Figure 6). 1 ng of DNA input was used because many DToL have low input into library preparation and therefore we could not afford to use higher quantities of DNA for a QC assay.

**TABLE 4:** Whole Genome Amplification (WGA) yield metrics and CCS yield for each test genome sample. Qubit: DNA concentration assessment using a Qubit fluorometer (Thermo Fisher Scientific, MA United States). TapeStation HS5000: DNA molarity assessment using the Agilent TapeStation using HS5000 tape (Agilent Technologies LDA UK Ltd., United Kingdom). TapeStation genomic tape: DNA molarity assessment using the Agilent TapeStation using genomic tape. Femto Pulse: DNA molarity assessment using the Agilent Femto Pulse (Agilent Technologies LDA UK Ltd., United Kingdom).

	CCS yield	DNA after WGA	TapeStation genomic tape			FemtoPulse
	Gb	qubit (ng/µl)	nM	nM 6kb+	ng/µl	ng DNA of size 6-60 kb
*Huperzia selago*	16	10.5	18.6	1.27	37.4	7.76
*Geum rivale*	8	11.3	1.1	0.01	2.68	0.0509
*Tholera decimalis*	9	12.5	57.1	4.63	111	37.2
*Lathraea squamaria*	17	3.53	13	0.902	23.6	6.58
*Adalia bipunctata*	21	22.7	46.2	4.5	109	40.4
*Biomphalaria glabrata*	0	1.12	4.89	0.131	7.22	0.839
*Dunaliella primolecta*	14	7.14	9.95	0.637	19.6	3.8
*Physelle acute*	7	8.03	13.1	0.758	24.1	4.53
*Quercus robur*	12	1.73	12.1	0.59	20.6	4.39
*Teleogryllus oceanicus*	20	3.88	19.1	1.01	33.9	6.47

Again, assay results were variable. Whilst *A. bipunctata* gave the highest WGA yield and the longest HMW DNA product on the Femto Pulse, the second best sample in terms of sequencing yield (*T. oceanicus*) gave only a moderate WGA yield and the observed fragments were smaller and fainter than other genomes (e.g., *Quercus robur* and *Tholera decimalis*) that gave much lower sequencing yield. The freshwater snail *Biomphalaria glabrata* DNA, however, that had failed HiFi sequencing yield also performed very poorly in this WGA assay. *Quercus robur*, gave low DNA yield, but produced a smear consisting of small and longer fragments, while *G. rivale* produced an average DNA yield, but produced only small fragments. Both of these had mediocre HiFi yield, showing some correlation to the WGA results.

**FIGURE 7:**
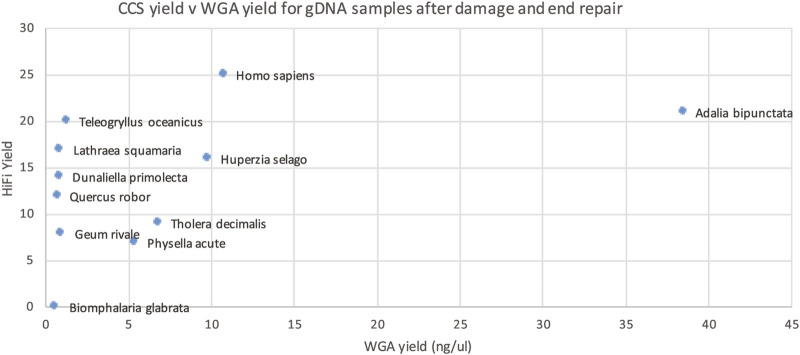
HiFi yield (Gb) versus whole genome amplification (WGA) yield (concentration in ng/ul for 50 ul) for each genomic DNA sample after PacBio library prep, including damage and end repair.

This WGA test however is performed before the damage repair step of PacBio library prep and thus cannot distinguish between damage that can, and cannot, be repaired. We reasoned that we might get a better correlation if we performed our WGA-based QC assay after the damage repair step (Figure 7). Though not perfect, this gave the best correlation of all our approaches, so we carried out a larger scale study on 200 samples.

**FIGURE 8:**
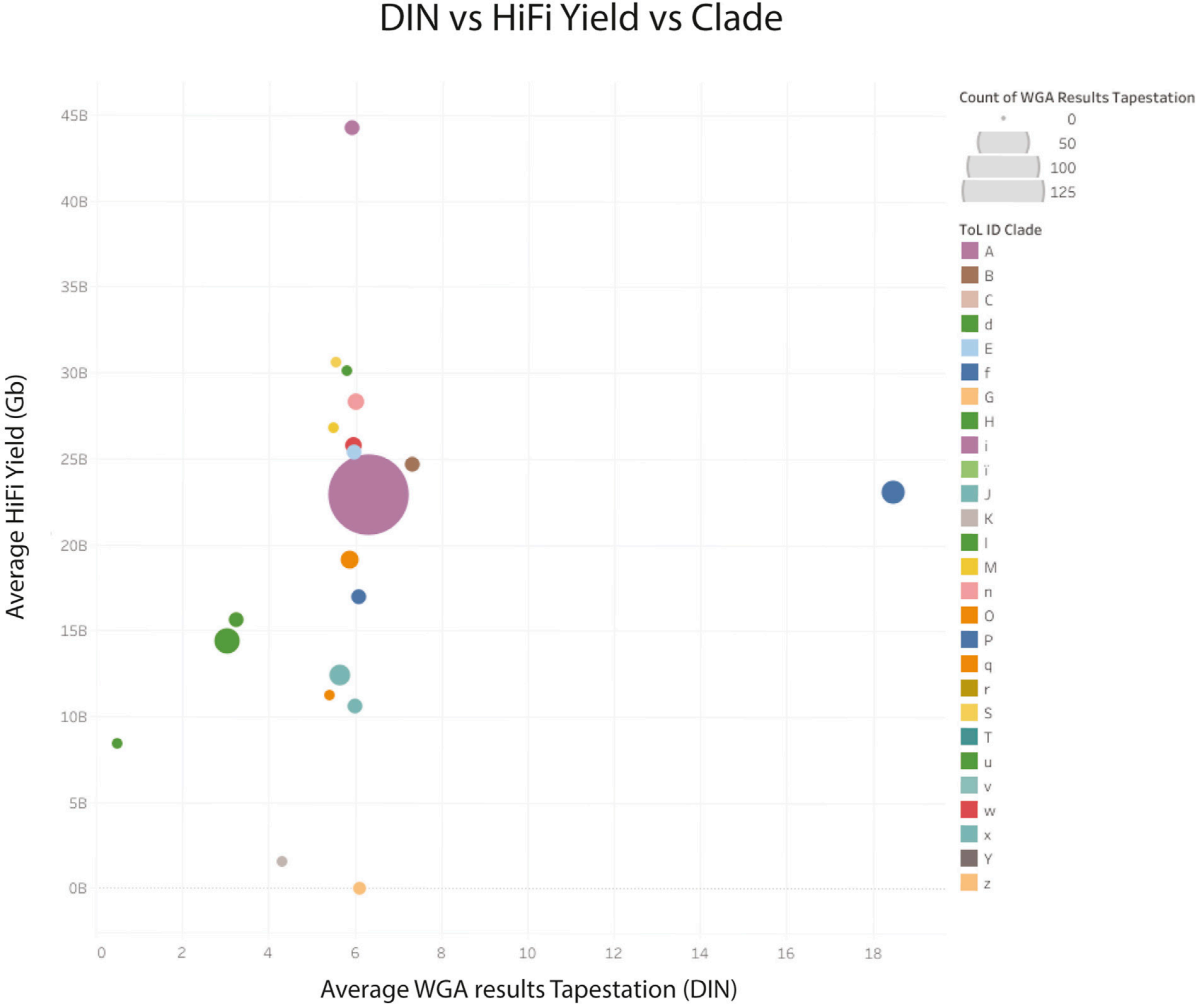
HiFi yield (Gb) versus whole genome amplification (DNA integrity number) yield for genomic DNA combined by clade. Clade identifiers can be found here (Tree of Life QC, 2024b). z Archaea; K, Other chordates and u: Algae, have very low CCS yields.

The larger dataset showed some samples with suboptimal sequencing and low amplification, or low DNA integrity numbers (DIN), however these metrics were not predictive of sequencing yield. The clade of the species was found to be more predictive of low yield (see Figure 8), however, the number of libraries sequenced for some clades was very low.

#### DNA Purity

We assayed the DToL R&D panel genomic DNA preparations for common biological compounds that could inhibit sequencing or reactions during library preparation. Specifically, we assayed for protein, RNA, carbohydrates, neutral lipids, cholesterol and polyphenols.

No protein or phenolics were detected with the available analytical methods (see materials and methods) in any of the samples. Lipid at 2.5 ng/μL could be detected in the *T. decimalis* gDNA but not in any of the other samples. Carbohydrates could be detected at 0.117 μg/μL in *A. bipunctata* and *Huperzia selago*, but both performed well in sequencing.

**FIGURE 9:**
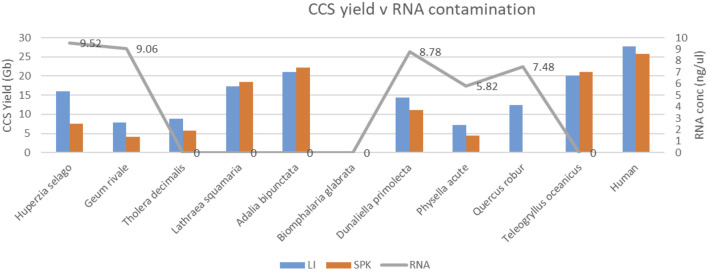
Relationship between HiFi yield (Gb) and RNA contamination (concentration in ng/ul; grey) in DNA extracts. For each species, extract DNA was split and subjected to two alternative library preparation protocols (LI; blue and SPK3.0 orange). Note that relatively low yielding species (<15 Gb on average) have detectable RNA contamination in five out of 7 cases.

Some gDNA preparations showed contamination with RNA (as measured with Qubit HS RNA kit [Thermo Fisher Scientific - United Kingdom Ltd.]), and the HiFi yields from sequencing runs that used those extracts were generally poor (Figure 9). However, no RNA could be detected in the sequencing libraries made from the RNA-contaminated gDNA (data not shown), so this weak correlation between yield and RNA-content of extracts may be spurious, or may be a indicator of other extraction co-contaminants we failed to detect.

Regardless of this inconclusive result, PacBio technical experts confirmed that RNA contaminants in the library could in principle interfere with the process of generating productive SMRT-bell complexes (although this does not appear to have been the case in our experiments). As a result of this we modified our protocols to i) verify the absence of RNA by including an RNA detection QC test after DNA extraction process (Qubit HS RNA kit [Thermo Fisher Scientific - United Kingdom Ltd.]) and ii) perform an extra RNAse digestion if RNA were detected.

### A modified SPRI clean-up method to remove fragments ≤5–10 kb

Our findings suggest that HiFi yields are lower when short DNA fragments, <5–10 kb, are present. These fragments are often generated during DNA extraction from challenging species and sample types and remain present in the final SMRTbell library. Circular Consensus Sequencing (CCS) is used to generate highly accurate long reads called HiFi reads. The polymerase needs to have sufficient passes of the SMRTbell template to build a consensus sequence with the required accuracy. In order to ensure sufficient passes in the sequencing run time, the desirable insert size for the template is ∼15–18 Kb (PacBio, 2021a). Shorter fragments, whilst generating high-quality data as a result of a higher number of passes, inhabit ZMWs for the duration of the run and therefore it is to be expected that short insert template will result in a reduced HiFi data yield.

The PacBio protocol, “Preparing HiFi Libraries from Low DNA Input Using SMRTbell Express Template Prep Kit 2.0” (from PacBio, CA, United States) includes a size selection using diluted AMPure PB Beads with Elution Buffer to 40% (v/v). Our results demonstrate this is insufficient for gDNA extractions with challenging size profiles, i.e., containing a large proportion of fragments under 10 Kb. Whilst techniques such as gel size selection are highly effective, requisite input amounts often exceed those available to us. Additionally, the resultant post-size selection yield may be insufficient for sequencing. Accordingly, to meet the requirements for a size selection technology capable of removing fragments up to 10 Kb (that is also tolerant to a wide range of input amounts typical of DToL samples, does not add complexity to laboratory workflows, is amenable to automation, and reliably recovers high yields of on target library fragments) we developed a “modified SPRI” approach (see Materials and Methods for detailed methodology) (Park, 2021).

**FIGURE 10:**
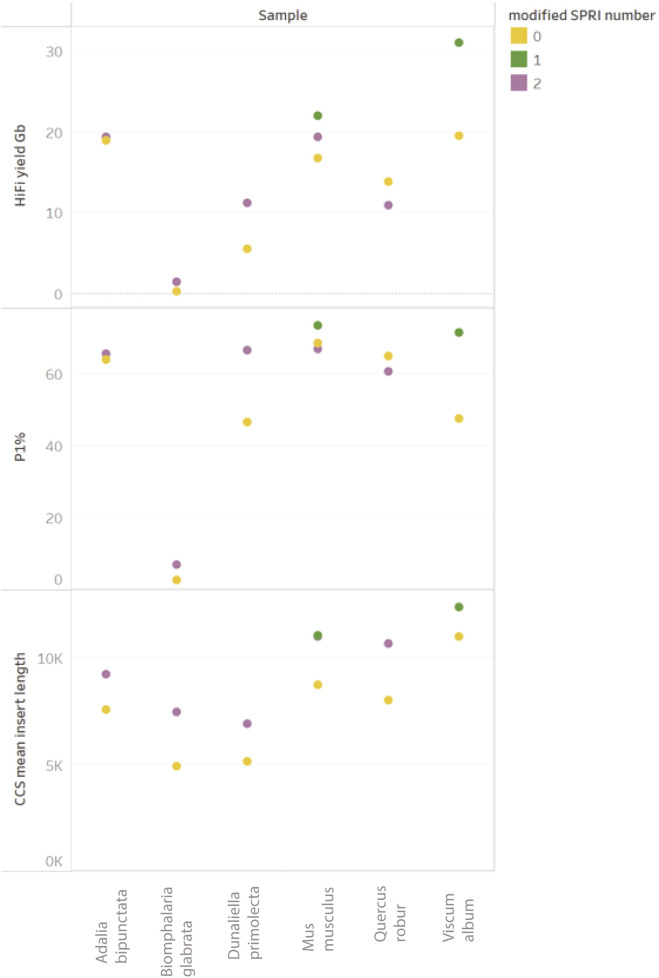
Comparison of ToL R&D panel species prepared with “Preparing HiFi Libraries from Low DNA Input Using SMRTbell Express Template Prep Kit 2.0” (from PacBio, CA, United States) and one DToL sample (Viscum album) prepared with “Preparing whole genome libraries using SMRTbell prep kit 3.0” protocol (from PacBio, CA, United States). Each sample (post shearing) was equally split between with modified-SPRI (2 = modified SPRI performed pre and post library construction (shown in purple), 1 = modified SPRI performed post library construction only (shown in green) and without any modified SPRI (shown in yellow).

The data obtained in our experiments (see Figure 10) shows a systematic increase in HiFi mean insert length when using the modified SPRI-clean up, as well as a corresponding change in the Femto Pulse electropherogram (Park, 2021), data in patent). An increase in P1% correlating to improved HiFi yields is observed for all samples with one notable exception (*Q. robur*) in which a slight reduction was observed. Improvements to HiFi yields due to modified SPRI use presented here are marginal due to the limited presence of fragments <10 kb in the input gDNA. However, since the introduction of this method in our operational library preparation pipeline, a clearer trend of improvement has been observed, in particular for samples containing significant molar amounts of fragments <5 kb. Whilst the presence of small DNA fragments can negatively impact yields, it is not the only factor contributing to low HiFi yields.

**FIGURE 11:**
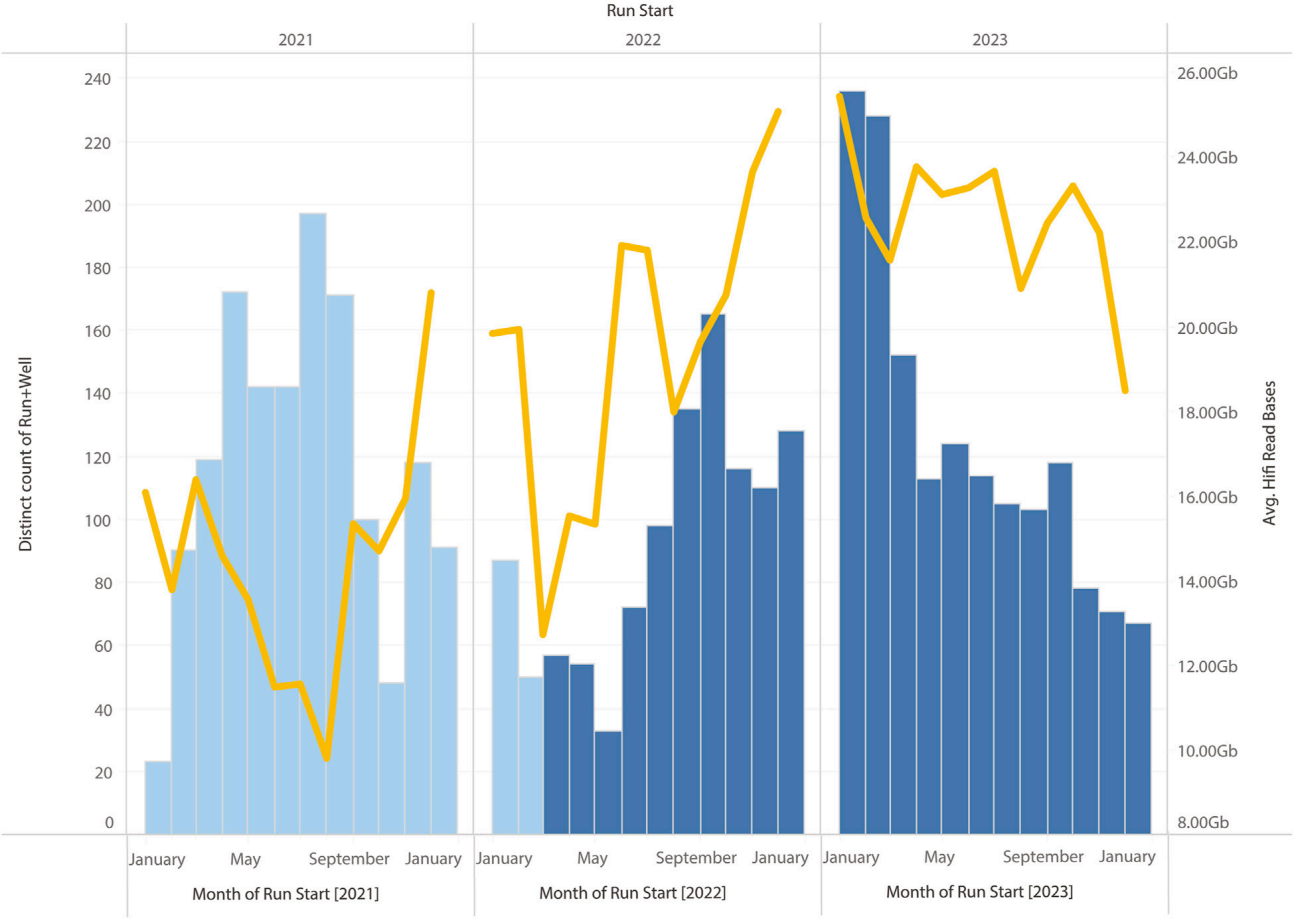
Monthly HiFi yields and number of SMRT Cells run on Sequel IIe. The modified SPRI was introduced during March 2022, other optimisations to the workflow, including change to the purification and shearing of samples, and the use of the SMRTbell prep kit 3.0 (from PacBio, CA, United States) were introduced in August 2022. A clear increase in average yield can be observed compared to samples run in 2022.

In summary, the changes to the library preparation process that have been tested have brought about systematic improvements to sequencing HiFi yields. However, other modifications to the library preparation process (e.g., changes to the shearing process) have also been implemented at the same time. This has made it difficult to clearly demonstrate HiFi yield improvements are due to the modified SPRI protocol alone. However, all these changes to the library preparation protocol have resulted in marginal gains, and their cumulative effect has translated into a systematic improvement over time (see Figure 11).

### Implementation of the Ultra Low Input sample preparation workflow

PacBio’s PCR based ultra-low input (ULI) library preparation protocol (PacBio, 2021b) has been developed to help sequence small organisms or samples where DNA yield from extraction is limited. This workflow reduces DNA quantity requirements down to 5 ng of sheared genomic DNA and relies on PCR amplification using two PCR enzymes to increase the DNA input for standard SMRTbell library preparation and sequencing. Because this kit uses PCR amplification, the DNA that is produced will be clean, unmodified DNA and we have seen high yields from this type of library (results not shown).

This methodology, however, has some drawbacks. PacBio recommends it is only suitable for genomes up to 0.5 Gb, (although we have gone up to 1 Gb, data not shown). In addition, both PCR enzymes exhibit some GC bias, which PacBio addresses by employing two differently biassed PCR enzymes. However, depending on input material and genome, this still does not always achieve uniform coverage (data not shown). Lastly, PCR amplification removes DNA modifications, which may limit ULI library preparation suitability for certain applications.

In addition to having the possibility to generate large amounts of DNA from limited input, we also have been using this methodology to generate higher HiFi yield from samples where native DNA generated a low sequencing yield. For instance, one group of fungal samples extracted using the commercial Nucleon PhytoPure extraction kit (Cytiva, United Kingdom), generated low sequencing yields, however sequencing yield could be reliably improved when 20 ng of sheared DNA from the same DNA extraction was subjected to the Ultra Low Input kit (results not shown). In some ineffective extraction methodologies, however, even the PCR enzymes within the Ultra-Low Input (ULI) kit show to be inhibited by some of the impurities co-purifying with the extracted DNA. Since the ULI library preparation protocol is routinely used to rescue suboptimal samples, we have seen this relatively frequently where one of the amplification reactions on the input material failed to produce amplified DNA (results not shown). In addition, we have observed that DNA extracts that originated from the same samples, but were extracted using different extraction methodologies, would fail amplification. We conclude that this inhibition of amplification is caused by differences in contaminant carry-over brought by ineffective cleaning of the isolated DNA, meaning DNA chelating, or DNA modifying contaminants would affect library yield, and not biological determinants, such as DNA methylation status or GC content. This again shows that carry-over, or potential cross linking of metabolites during inefficient extraction processes can interfere with DNA, hampering its ability to sequence or amplify.

## Discussion

In this paper, we detail our work investigating the drivers of PacBio HiFi yield variability which we observed when sequencing a diverse and challenging set of DNA samples, primarily submitted by the Tree of Life Programme at the Wellcome Sanger Institute.

We have shown that limited yield variability could be attributed to the PacBio sequencing system and the library preparation methodologies used for generating suitable sequencing libraries, especially when using high quality DNA.

We could not identify a single root cause for variable HiFi yields. DNA damage, contamination with impurities inhibiting the adaptor ligation and/or DNA polymerase, and the presence of short DNA fragments all contribute to varying degrees. However, we have not been able to identify a single assay that can be used to predict sequencing outcome. Given the cost of sequencing and the potential value of samples from some species, coupled with the yield variability observed in non-model samples, and the expansion of sequencing consortia focusing on biodiversity, there is growing urgency and demand for such an assay(s).

We observe that the inclusion of a PCR pre-amplification step during the library preparation generates material that routinely results in high-yielding sequencing runs. Applying this amplification methodology to samples that had poor sequencing results when using amplification-free methods shows that it is the quality of the DNA, and based upon Sanger’s ULI rescued genomes, not the DNA sequence, that is the cause of the poor sequencing yield. Although we do not advocate amplification for all samples, we have observed that the quality of the ULI sequencing data is adequate for genome assemblies if combined with data from standard amplification-free libraries. This is especially true when the genome size is within PacBio’s recommended specifications of 0.5 Gb, however our experiments have shown that this can work for species with a genome size of up to 1 Gb (results not shown).

We conclude, therefore, that a primary factor driving the level of variability we observed for DToL samples (between 0 and 38 Gb) is the quality of the DNA prior to library construction, e.g., purity, size, and damage.

With these conclusions in mind, further work is required to:

• Identify sample extraction methods adapted to the requirements of different taxa and the development of suitable standardised protocols, to avoid DNA damage and carry over of potential contaminants.
• Develop quality control assays which can predict PacBio sequencing outcomes with high accuracy and sensitivity. As we have shown, some assays correlate to some extent with low sequencing yield, however no single assay described in this work is adequate for predicting sequencing success for all sample types due to the multifactorial nature of the drivers behind the variability.
• Develop a limited-cycle pre-amplification (modified ULI workflow) library preparation method, in an effort to generate higher quality data for samples which cannot be sequenced using amplification-free library preparation methods. These developments would aim to reduce PCR-based sequence bias.

The Darwin Tree of Life project, and the Earth BioGenome project are just beginning, and it is clear that, as a community, we need to develop solutions to the challenges we have described in this manuscript. We hope that researchers and sequencing core facilities will use this as a foundational resource and build upon our investigative work to find suitable purification methods and quality control assays to increase sequencing success for samples that are currently difficult to sequence.

## Materials and methods

### Run monitoring

With the adoption of the Sequel IIe system within our institute, run performance was tracked by recording key metrics, e.g., Sequencing yield, read N50, read length distribution, number of control reads, etc. When comparing HiFI yields with quality control metrics (e.g., average DNA size, presence of small fragments, control reads), we observed a number of correlating metrics for some samples, e.g., low read counts for the PacBio internal control complex, and low P1 percentage (SMRT Cell productivity metric).

### DNA preparation

Most of the DNA samples described in this paper were extracted using the Qiagen Magattract HMW kit (Cat. No. 67563) according to the 10X modified Magattract protocol (DNA Extraction from Blood Protocol Revision B; 10X Genomics).

### PacBio library preparation protocols

Unless stated otherwise PacBio libraries were prepared using the “Preparing HiFi Libraries from Low DNA Input Using SMRTbell Express Template Prep Kit 2.0” protocol (from PacBio, CA, United States).

Where indicated, PacBio libraries were prepared using the “Preparing whole genome libraries using SMRTbell prep kit 3.0” protocol (from PacBio, CA, United States).

### Library control

The library control was run 13 times over a 9-week period. All library controls passed library preparation. The pass/fail criteria are based on the maximum on plate loading concentration (OPLC) that could be achieved for 1 SMRT Cell assuming 50% recovery from the ABC reactions. A “fail” is a sample where the maximum OPLC falls below 30 pM.

### DNA integrity assays

#### PCR assay

20 µL DNA at ∼1 ng/μL was sheared to approximately 10 kb using a Diagenode Megaruptor 3 on speed setting 46, prior to end prep, A-tailing, ligation to Xgen stubby adapter (IDT cat no. 10005924) and 0.9x Ampure XP cleanup. 0.5 ng of adapter ligated DNA was used as template for long PCR with either LA Taq DNA polymerase (Takara Bio Europe, France) or RepliQa HiFi ToughMix (Quantabio, MA, United States) in the presence of premixed IDT UDI indexing primers (IDT cat. no. 10005975). Cycling conditions were: 1 µM primers. LAtaq: 94°C for 1 min, 16 cycles of 98°C for 10 s, 60°C for 15 s, 68°C for 20 min. RepliQa: 98°C for 30 s, 16 cycles of 98°C for 10 s, 60°C for 15 s, and 68°C for 10 min. Post PCR amplified products were purified using Ampure XP at a 0.7:1 ratio (i.e., 35 μL of Ampure XP beads plus 50 µL of PCR product) and eluted in 30 μL EB elution buffer (QIAGEN). Amplification products were quantified using Qubit dsDNA Quantitation, broad range Assay kit (Cat. no. Q32853) according to the manufacturer’s protocol.

#### qPCR assay

20 µL DNA at ∼1 ng/μL was sheared to approximately 10 kb using a Diagenode Megaruptor 3 on speed setting 46, prior to end prep, A-tailing, ligation to IDT for Illumina Truseq UD adapters (Illumina cat. no. 200408700) and 0.9x Ampure XP cleanup. 0.1 ng adapter-ligated DNA was used as template for long cycle qPCR (extension at 68°C for 20 min) with either LA taq (Takara) or RepliQa (Quantabio) in presence of 1 µM P5 and P7 primers (AAT GAT ACG GCG ACC ACC GA and CAA GCA GAA GAC GGC ATA CGA), 1 µM ROX (thermo cat no. 12223012) and 1 in 500 diluted SYBR green nucleic acid stain (Merck cat. no. S9430-1 ML) in an ABI StepOne plus qPCR instrument. Amplification based Ct values were calculated by the instrument.

#### Nicking assay

20 ng of DNA was incubated at 23°C for 20 min in 1x S1 buffer with 100 units of Takara S1 nuclease (cat no. 2410B) in a total volume of 50 μL, after which the nick digest assay was terminated by adding 10 µL of 100 mM EDTA. The reaction was purified by performing a 2x Ampure XP cleanup and eluted in 30 μL EB buffer, diluted to 0.25 ng/μL and DNA fragment sizes analysed by electrophoresis on Agilent Femto Pulse Genomic DNA 165 kb kit (cat. no. FP-1002-0275). Fragment size distributions were compared to those obtained prior to S1 nuclease digestion.

#### Whole Genome Amplification (WGA) assay

WGA, using a phi29 polymerase based amplification, was performed using the Qiagen Repli-G ultrafast mini kit (cat no. 150033). 1 ng DNA in a volume of 1 µL was denatured and amplified according to manufacturer’s instructions. WGA products were analysed by running a 1 µL aliquot on an Agilent TapeStation Genomic DNA Assay (cat no. 5067–5365) and by quantifying the amount of DNA produced using the Qubit dsDNA Quantitation, broad range Assay kit (Cat. no. Q32853).

### Chemical contamination assays

#### RNA

RNA contamination of DNA preps was tested using the Invitrogen Qubit RNA high sensitivity assay kit (cat. no. Q32852) according to manufacturer instructions.

#### Protein

Protein contamination of DNA preps was tested using the Invitrogen Qubit protein assay kit (cat. no. Q33211) according to manufacturer instructions.

#### Carbohydrate

Carbohydrate contamination of DNA preps was tested using the Total Carbohydrate Assay Kit - Quantification (Abcam cat. no. ab155891) according to manufacturer instructions.

#### Lipids

Neutral Lipid contamination of DNA preps was tested using the Lipid Assay Kit (neutral lipids) (Abcam cat. no. ab242307) according to manufacturer instructions.

#### Cholesterol

Cholesterol contamination of DNA preps was tested using the Cholesterol Assay Kit - HDL and LDL/VLDL (Abcam cat. no. ab65390) according to manufacturer instructions.

#### Polyphenols

Polyphenol contamination of DNA preps was tested using the Abcam Phenolic Compounds Assay Kit (Colorimetric) (cat. no. ab273293) according to manufacturer instructions.

### Modified SPRI for size selection

A modified SPRI solution was made containing 10% (w/v) PEG 6000, 1.9M NaCl, 1 mM EDTA, 10 mM Tris-HCl pH 8.0 in nuclease free water. 28 mL of AMPure XP beads were washed 4x with 28 mL of nuclease free water. A further wash with 28 mL of Elution Buffer (QIAGEN) preceded equilibration of the beads into 2 mL of modified SPRI solution. Finally, the washed beads were resuspended in 26.5 mL of modified SPRI solution to form the “modified SPRI beads” and were stored at 4°C. Each wash step was carefully performed to minimise bead loss throughout. The modified SPRI beads were combined at 0.98X, 0.97X, 0.96X, 0.95X ratios with sheared DNA to calibrate each batch and determine the working ratio to obtain a consistent 7 kb cutoff, by running the size selected DNA on an Agilent Femto Pulse. This protocol has been patented and can be found under patent application number: PCT/GB2023/052127. Please contact authors for further details.

### PacBio Ultra Low Amplification library prep

PacBio Ultra Low Amplification libraries were made according to PacBio’s protocol from 20 ng of input material with eight cycles of PCR amplification each. After size estimation on the Femto Pulse using the 55 kb BAC Analysis Kit, size selection was done using the BluePippin (Sage Science, MA, United States) at appropriate sizes (5–8 kb) to include at least 60% of the peak after size selection. Pooling of the samples was based on molarity to get equimolar pools.

## Data Availability

The datasets presented in this article are not readily available because the meta-analysis of the sequencing QC data is highlighted in the manuscript. We did not further use generated sequencing data since this was out of scope for this manuscript. Requests to access the datasets should be directed to iraad.bronner@sanger.ac.uk.

## Supplementary material

SUPPLEMENTARY FIGURE 1 DNA recovery for library controls and samples on associated library preparation batches. On the left, DNA recovery for library preparation (excluding the size selective SPRI) shown as a percentage. On the right, DNA Recovery for the size selective SPRI shown as a percentage. Colour shows detail about sample.

SUPPLEMENTARY FIGURE 3 Average HiFi Yield (Gb) for each genomic quality number (GQN) bin. **(A)** shows GQN after DNA extraction. Colour shows detail about whether the GQN value is≥5 when using a threshold of 30 Kb (PacBio recommends samples should have a GQN≥5 after DNA extraction for optimal sequencing results). **(B)** shows GQN after shearing. Colour shows detail about whether the GQN value is≥9 when using a threshold of 10 Kb (PacBio recommends samples should have a GQN≥9 after shearing for optimal sequencing results). Each circle represents one DNA extraction. Multiplexed samples are not shown.
