# Supplementary material for: Evaluation of controls, quality control assays, and protocol optimisations for PacBio HiFi sequencing on diverse and challenging samples
Source: Front Genet. 2025 Jan 6;15:1505839. doi: 10.3389/fgene.2024.1505839 (PMC11752452; doi:10.3389/fgene.2024.1505839)
Supplement: Supplementary file 1 [file Table1.docx]

| **Taxon** | **Species** | **Common name** | **Genome size (Mb)** | **Taxon ID** | **Ploidy** | **Part of R&D panel** | **Link to ToLQC data** |
| --- | --- | --- | --- | --- | --- | --- | --- |
| Mollusc | *Biomphalaria glabrata* |  | 929 | 6526 | 2 | yes | <https://tolqc.cog.sanger.ac.uk/tol/molluscs/Biomphalaria_glabrata/> |
| Fungi | *Metschnikowia zobellii* |  | 13 | 27328 | 12 | yes | <https://tolqc.cog.sanger.ac.uk/darwin/fungi/Metschnikowia_zobellii/> |
| Plant | *Geum rivale* | Water avens | 1261 | 148897 | 12 | no | <https://tolqc.cog.sanger.ac.uk/darwin/dicots/Geum_rivale/> |
| Plant | *Lathraea squamaria* | Common toothwort | 1378 | 374711 | 4 | no | <https://tolqc.cog.sanger.ac.uk/darwin/dicots/Lathraea_squamaria/> |
| Arthropod | *Adalia bipunctata* | Two-spot ladybird, two-spotted ladybug or two-spotted lady beetle | 352 | 7084 | 2 | yes | <https://tolqc.cog.sanger.ac.uk/darwin/insects/Adalia_bipunctata/> |
| Algae | *Dunaliella primolecta* |  | 276 | 257627 | 38 | yes | <https://tolqc.cog.sanger.ac.uk/darwin/algae/Dunaliella_primolecta/> |
| Plant | *Quercus robur* | Pedunculate oak or English oak | 1017 | 38942 | 2 | yes | <https://tolqc.cog.sanger.ac.uk/darwin/dicots/Quercus_robur/> |
| Arthropod | *Tholera decimalis* | Feathered gothic | 1320 | 988041 | 2 | no | <https://tolqc.cog.sanger.ac.uk/darwin/insects/Tholera_decimalis/> |
| Plant | *Huperzia selago* | Northern firmoss or fir clubmoss | 4938 | 70001 | 2 | no | <https://tolqc.cog.sanger.ac.uk/darwin/vascular-plants/Huperzia_selago/> |
| Mollusc | *Physella acuta* | European physa, tadpole snail, bladder snail, and acute bladder snail | 702 | 109671 | 2 | no | <https://tolqc.cog.sanger.ac.uk/darwin/molluscs/Physella_acuta/> |
| Chordate | *Mus musculus* | House mouse | 3198 | 10090 | 2 | yes | <https://tolqc.cog.sanger.ac.uk/darwin/mammals/Mus_musculus/> |
| Arthropod | *Teleogryllus oceanicus* | Australian, Pacific or  oceanic field cricket | 2028 | 128161 | 6 | yes | <https://tolqc.cog.sanger.ac.uk/darwin/insects/Teleogryllus_oceanicus/> |

Supplementary Figure 2. Overview of species directly mentioned in the article text, with species name, common name (when available), genome size, Taxon ID and ToLQC reported ploidy level (i.e. 2 for diploid). In addition, it is highlighted whether samples are part of our routine R&D panel or not. The last column is where ToLQC data can be found on the internet.
