# Supplementary figures and images for: Evaluation of controls, quality control assays, and protocol optimisations for PacBio HiFi sequencing on diverse and challenging samples
Source: Front Genet. 2025 Jan 6;15:1505839. doi: 10.3389/fgene.2024.1505839 (PMC11752452; doi:10.3389/fgene.2024.1505839)

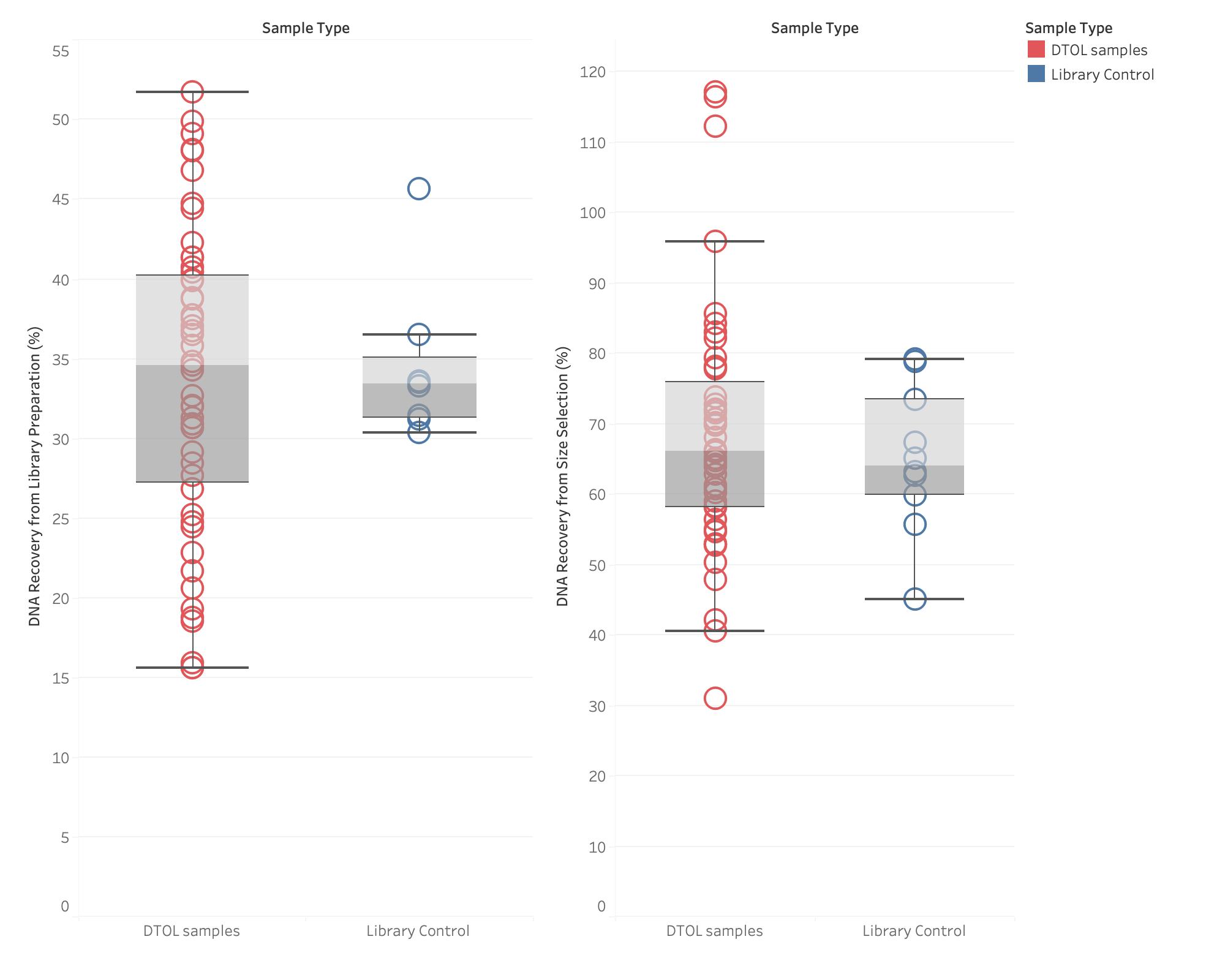

Supplement: Supplementary file 2 [file Image1.jpeg]

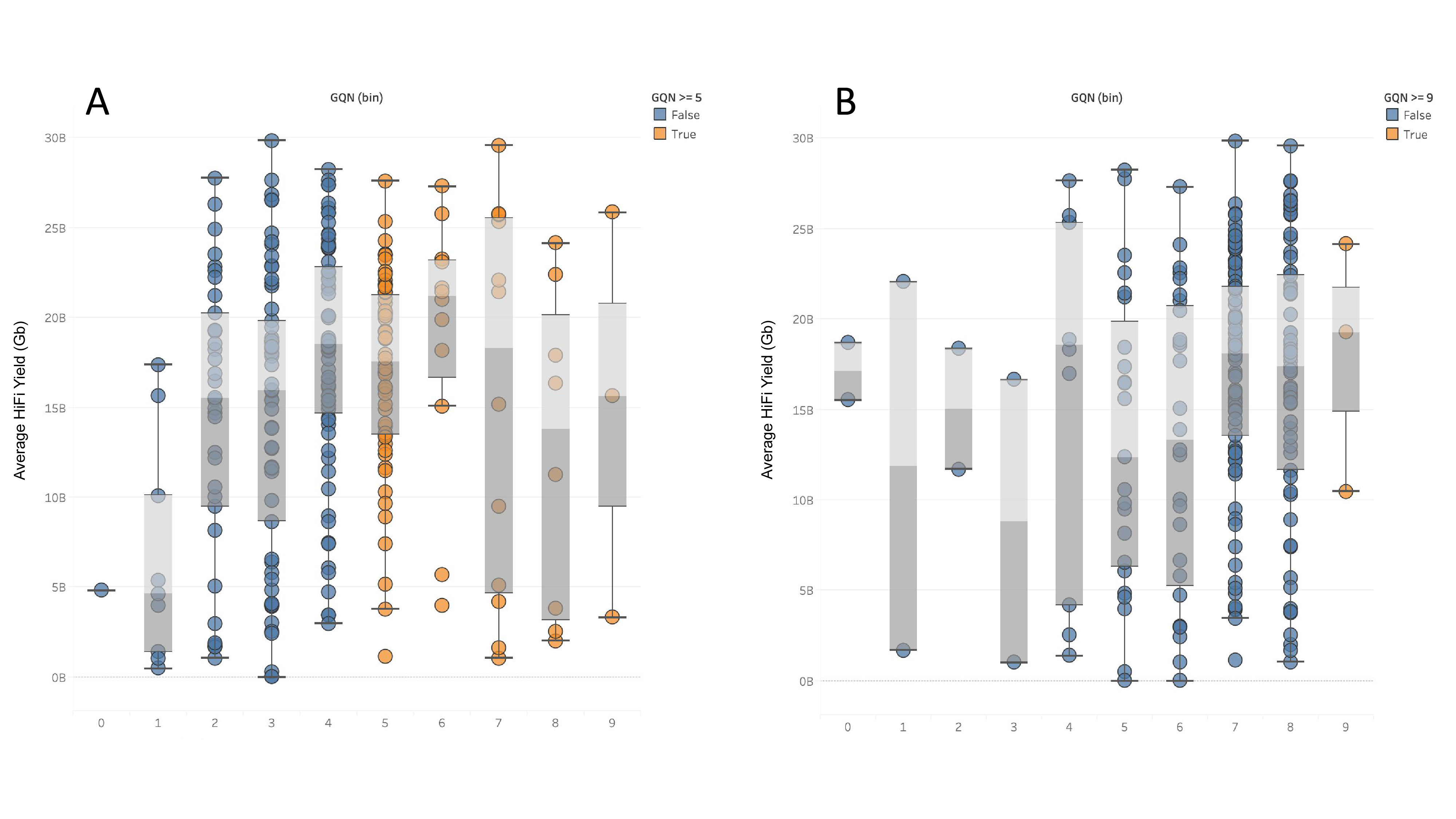

Supplement: Supplementary file 3 [file Image2.jpeg]
